# Decision-making at the limit of viability: differing perceptions and opinions between neonatal physicians and nurses

**DOI:** 10.1186/s12887-018-1040-z

**Published:** 2018-02-22

**Authors:** Hans Ulrich Bucher, Sabine D. Klein, Manya J. Hendriks, Ruth Baumann-Hölzle, Thomas M. Berger, Jürg C. Streuli, Jean-Claude Fauchère, on behalf of the Swiss Neonatal End-of-Life Study Group, Meyer Philipp, Neumann Roland, Itin Renate, Nelle Mathias, Stoffel Liliane, Scharrer Brigitte, Roloff Kai, Pfister Riccardo, Roth Matthias, Contino Magali, Berger Thomas, Schlegel Ulrike, Jaeger Gudrun, Dutler Ruth, Fauchère Jean-Claude, Dinten Barbara

**Affiliations:** 1 Department of Neonatology, University Hospital Zurich, Frauenklinikstrasse 10, 8091 Zürich, Switzerland; 2 Institute of Biomedical Ethics and History of Medicine, University of Zurich, Zurich, Switzerland; 3 Dialogue Ethics Foundation, Interdisciplinary Institute for Ethics in Health Care, Zurich, Switzerland; 4 Neonatal and Paediatric Intensive Care Unit, Children’s Hospital of Lucerne, Lucerne, Switzerland

**Keywords:** Extremely preterm infants, Neonatal intensive care, End-of-life decisions, Shared decision-making, Health care professionals, Previable period

## Abstract

**Background:**

In the last 20 years, the chances for intact survival for extremely preterm infants have increased in high income countries. Decisions about withholding or withdrawing intensive care remain a major challenge in infants born at the limits of viability. Shared decision-making regarding these fragile infants between health care professionals and parents has become the preferred model today. However, there is an ongoing ethical debate on how decisions regarding life-sustaining treatment should be reached and who should have the final word when health care professionals and parents do not agree. We designed a survey among neonatologists and neonatal nurses to analyze practices, difficulties and parental involvement in end-of-life decisions for extremely preterm infants.

**Methods:**

All 552 physicians and nurses with at least 12 months work experience in level III neonatal intensive care units (NICU) in Switzerland were invited to participate in an online survey with 50 questions. Differences between neonatologists and NICU nurses and between language regions were explored.

**Results:**

Ninety six of 121 (79%) physicians and 302 of 431(70%) nurses completed the online questionnaire. The following difficulties with end-of-life decision-making were reported more frequently by nurses than physicians: insufficient time for decision-making, legal constraints and lack of consistent unit policies. Nurses also mentioned a lack of solidarity in our society and shortage of services for disabled more often than physicians. In the context of limiting intensive care in selected circumstances, nurses considered withholding tube feedings and respiratory support less acceptable than physicians. Nurses were more reluctant to give parents full authority to decide on the course of action for their infant. In contrast to professional category (nurse or physician), language region, professional experience and religion had little influence if any on the answers given.

**Conclusions:**

Physicians and nurses differ in many aspects of how and by whom end-of-life decisions should be made in extremely preterm infants. The divergencies between nurses and physicians may be due to differences in ethics education, varying focus in patient care and direct exposure to the patients. Acknowledging these differences is important to avoid potential conflicts within the neonatal team but also with parents in the process of end-of-life decision-making in preterm infants born at the limits of viability.

**Electronic supplementary material:**

The online version of this article (10.1186/s12887-018-1040-z) contains supplementary material, which is available to authorized users.

## Background

Over the past decades, survival of extremely preterm infants has improved with new technological, medical and neonatal care developments [1]. However, deciding to initiate, withhold or withdraw intensive care for infants born at the limit of viability remains a difficult decision in modern neonatal medicine [2]. According to a recent retrospective cohort study of infants born between 22 and 27 completed weeks of gestation in Switzerland, a decision to withhold active treatment before or immediately after birth is taken only for a minority of these infants [3]. Instead, most infants born ≥24 weeks are offered provisional intensive care. Most extremely preterm infants who die in neonatal intensive care units (NICUs) do so after a decision to redirect treatment from intensive care to comfort care (i.e., withdrawal of life-sustaining therapies) [4, 5].

Today, it is generally accepted that end-of-life decisions in extremely preterm infants should be made in a process involving physicians, nurses and parents [6, 7]. This makes it especially important to discuss and reflect upon the – possibly – diverse moral attitudes and values of stakeholders, and specifically in this study, health care professionals.

It has been shown that mortality rates adjusted for gestational age, sex and other risk factors vary widely between hospitals and between countries [8, 9]. Moreover, end-of-life decisions are not only based on outcome statistics but are greatly influenced by the attitudes, values and perceptions of the reported outcomes by the different parties involved in this decisional process [10].

We were interested to explore the kind of problems that neonatologists and NICU nurses identify in end-of-life decision-making and how they perceive the role of parents in this process. For this reason, an online survey was performed among all nine level III NICUs in Switzerland. We also aimed at investigating whether the answers were associated with professional status, years of professional experience, importance of religion or language region.

## Methods

A questionnaire was prepared integrating selected items from a previously used questionnaire [11] and also questions used in a telephone survey of a representative sample of the Swiss population [12]. The initial English questionnaire was translated into German and French. The translation accuracy was checked and reviewed by a panel of translators to ensure identical semantic content in each language.

The questionnaire consisted of 32 statements about end-of-life decision-making in infants born before 28 weeks’ gestation, nine statements about prenatal decision-making (for physicians only), five questions regarding dissent between parents and health care professionals (HCPs) and about the role of a Hospital’s Ethics Committee, each with several options, from which only one could be chosen. Four questions were about professional education and experience and importance of religion of the participants (full questionnaire see Additional file 1).

The goals of this study were presented to the staff of all nine Level III NICUS in Switzerland. All physicians and nurses who had been working for more than 12 months in a NICU setting (*n* = 552) were invited per e-mail to participate in an online survey. Participation was voluntary, interviewees were asked for consent preceding the actual online survey. Non-respondents received two reminders. Data were anonymised before analysis. No formal approval of this survey was required by the Ethics Committee of the Canton Zurich.

Statistical analysis was performed using IBM SPSS Statistics 22 (Armonk, NY, USA). The following groups were compared using chi-squared, Kruskal-Wallis or Mann-Whitney U tests: physicians vs. nurses, German vs. French speaking areas, duration of professional experience (≤ 5, 6–15, > 15 years), importance of religion (important vs. not important). Results are presented as proportions and 95% confidence intervals (Wilson score intervals). Significance was defined as *p* < 0.05.

## Results

Ninety six of 121 (79%) physicians and 302 of 431 (70%) nurses completed the online questionnaire. The characteristics of the participants are given in Table 1. Compared to nurses, participating physicians were more frequently in a leading position and more often had children of their own.

**Table 1:** Characteristics of the survey participants

	Physicians		Nurses		Total	
	N	%	N	%	N	%
Language region						
German	74	77.1	210	69.5	284	71.4
French	22	22.9	92	30.5	114	28.6
Gender						
Men	47	50.0	19	6.3	66	16.7
Women	47	50.0	283	93.7	330	83.3
Age, yrs						
< 30	4	4.3	53	17.5	57	14.4
30–39	38	40.4	126	41.7	164	41.4
40–49	30	31.9	78	25.8	108	27.3
≥50	22	23.4	45	14.9	67	16.9
Experience in NICU, yrs						
≤3	26	27.1	49	16.2	75	18.8
4–6	18	18.8	66	21.9	84	21.1
7–10	14	14.6	58	19.2	72	18.1
11–20	29	30.2	84	27.8	113	28.4
> 20	9	9.4	45	14.9	54	13.6
Leading position						
Yes	87	90.6	47	15.6	134	33.7
No	9	9.4	255	84.4	264	66.3
Religious background						
None	23	24.5	85	28.1	108	27.3
Catholic	40	42.6	126	41.7	166	41.9
Protestant	26	27.7	70	23.2	96	24.2
Other	5	5.3	21	7.0	26	6.6
Importance of religion						
Important	30	31.9	96	31.8	126	31.8
Not important	64	68.1	206	68.2	270	68.2
Own children						
Yes	58	61.7	154	51.0	212	53.5
No	36	38.3	148	49.0	184	46.5

### Difficulties encountered in end-of-life decision-making

A large majority of the respondents indicated a range of problems when decisions about limiting intensive care for an extremely preterm baby must be made (Table 2). 94% of all HCPs found it difficult to foresee the patient’s future quality of life, and 90% named the difficulty to make an accurate long-term prognosis. The following difficulties with end-of-life decision-making were reported more frequently by nurses than physicians: difficulty of interpreting parents’ attitudes precisely (92% vs. 82%), insufficient time for decision-making (81% vs. 54%), legal constraints (80% vs. 54%), lack of a consistent unit policy (73% vs. 36%) and conflict between your own principles and unit policy (60% vs. 40%). In comparison to physicians, nurses also mentioned significantly more often a lack of solidarity in our society (46% vs. 22%) and a shortage of services for disabled (46% vs. 12%). Lack of a consistent unit policy was more often indicated in the French speaking area (81%) than in the German speaking area (58%).

**Table 2:** Important problems when making decisions about limiting intensive care

Question	Total	Physicians	Nurses	*p*-value	German speaking area	French speaking area	*p*-value
Difficulty in foreseeing patient’s future quality of life	93.8 (91.0–95.8)	94.7 (88.3–97.7)	93.5 (90.1–95.8)	0.673	92.8 (89.1–95.3)	96.4 (91.1–98.6)	0.184
Difficulty of making an accurate long term prognosis	89.5 (86.1–92.2)	92.6 (85.6–96.4)	88.6 (84.4–91.7)	0.259	89.2 (85.1–92.4)	90.3 (83.4–94.5)	0.766
Difficulty of interpreting parents’ attitudes precisely	89.3 (85.8–92.0)	81.7 (72.7–88.3)	91.7 (88.0–94.4)	0.007	88.3 (84.0–91.6)	91.7 (85.0–95.6)	0.329
Insufficient time for decision-making	74.2 (69.6–78.4)	54.3 (44.2–64.0)	80.9 (75.9–85.0)	< 0.001	74.7 (69.3–79.5)	72.8 (63.5–80.5)	0.706
Legal constraints	73.3 (68.6–77.6)	54.3 (44.2–64.1)	79.5 (74.4–83.8)	< 0.001	74.0 (68.4–78.9)	71.8 (62.8–79.4)	0.669
Impossibility of obtaining the patient’s own views	66.2 (61.2–70.9)	64.9 (54.8–73.8)	66.7 (60.9–72.0)	0.754	69.3 (63.5–74.6)	58.3 (48.6–67.3)	0.044
Lack of a consistent Unit policy to guide you	64.4 (59.3–69.2)	36.1 (26.6–46.9)	73.0 (67.4–77.9)	< 0.001	58.1 (52.0–64.0)	80.8 (72.0–87.4)	< 0.001
Conflict between your own principles and Unit policy	55.5 (50.4–60.5)	40.0 (30.5–50.3)	60.4 (54.6–65.9)	0.001	53.5 (47.6–59.4)	60.6 (51.0–69.4)	0.220
Difficulty of foreseeing future developments in medicine which may help babies who now appear hopeless cases	43.2 (38.2–48.3)	29.8 (21.5–39.7)	47.8 (41.9–53.7)	0.002	43.9 (38.1–49.8)	41.2 (32.0–51.2)	0.654
Society’s lack of solidarity for the disabled	40.4 (35.6–45.4)	22.1 (14.9–31.4)	46.4 (40.7–52.1)	< 0.001	39.7 (34.1–45.6)	42.2 (33.4–51.6)	0.654
Shortage of services for the disabled	37.5 (32.7–42.6)	12.4 (7.0–20.8)	45.5 (39.8–51.4)	< 0.001	33.7 (28.3–39.7)	46.7 (37.6–56.1)	0.019

Answers to the question: “How important do you consider each of the following problems when making decisions about whether or not to limit intensive care for an extremely preterm baby?” Percentages of respondents who answered “very important” or “important” with 95% confidence intervals are shown. Answers are listed in decreasing importance. Total *n* = 397

### Acceptable approaches to limiting intensive care

Administering sedatives and/or analgesics to suppress pain even if this might cause respiratory depression and death was acceptable to 95% of all respondents (Table 3). 24% accepted administering drugs with the explicit purpose to hasten death. Compared to physicians, nurses significantly less often indicated that withholding intensive care (83% vs. 100%), refraining from increasing respiratory support (66% vs. 80%) and withholding full parenteral nutrition (50% vs. 75%), tube feeding (28% vs. 45%) or antibiotics (67% vs. 80%) would be acceptable options when it comes to limiting intensive care.

**Table 3:** Acceptable approaches of limiting intensive care

	Total	Physicians	Nurses	*p*-value	German speaking area	French speaking area	*p*-value
Administering sedatives and/or analgesics to suppress pain even if this might cause respiratory depression and death	95.1 (92.4–96.8)	96.8 (91.0–98.9)	94.5 (91.2–96.6)	0.367	94.9 (91.5–96.9)	95.5 (90.0–98.1)	0.779
Withholding emergency treatment/manoeuvres	94.9 (92.2–96.6)	100.0 (96.1–100.0)	93.2 (89.7–95.6)	0.009	93.9 (90.4–96.1)	97.3 (92.4–99.1)	0.162
Withholding surgery	94.0 (91.1–96.0)	100.0 (96.0–100.0)	92.0 (88.3–94.6)	0.005	93.0 (89.3–95.5)	96.4 (91.1–98.6)	0.204
Withdrawing life-saving drugs	93.5 (90.6–95.6)	97.9 (92.6–99.4)	92.1 (88.4–94.7)	0.048	93.5 (89.9–95.8)	93.6 (87.3–96.9)	0.965
Withdrawing mechanical ventilation	91.2 (87.9–93.6)	97.8 (92.4–99.4)	89.1 (85.0–92.2)	0.010	90.9 (86.9–93.7)	91.9 (85.3–95.7)	0.751
Continuing current treatment, but without adding others	89.6 (86.1–92.3)	98.9 (93.9–99.8)	86.7 (82.3–90.2)	0.001	87.5 (83.0–91.0)	94.6 (88.7–97.5)	0.040
Withholding intensive care	87.4 (83.7–90.4)	100.0 (96.1–100.0)	83.3 (78.6–87.2)	< 0.001	84.6 (79.9–88.4)	94.5 (88.5–97.5)	0.009
Withholding antibiotics	70.6 (65.8–75.0)	80.4 (71.2–87.3)	67.4 (61.7–72.6)	0.017	69.8 (64.0–75.0)	72.6 (63.5–80.2)	0.589
Refraining from increasing the respirator parameters	69.7 (64.9–74.1)	80.2 (70.9–87.1)	66.3 (60.6–71.6)	0.012	66.2 (60.2–71.6)	78.2 (69.6–84.9)	0.021
Withholding full parenteral nutrition	55.9 (50.7–60.9)	75.0 (65.3–82.7)	49.5 (43.6–55.3)	< 0.001	54.4 (48.3–60.3)	59.4 (49.9–68.3)	0.380
Withholding tube feeding	31.9 (27.3–36.8)	45.2 (35.0–55.9)	27.9 (22.9–33.4)	0.003	33.6 (28.1–39.5)	27.5 (19.7–36.8)	0.260
Administering drugs with the explicit purpose to hasten death	24.4 (20.2–29.2)	18.2 (11.5–27.5)	26.5 (21.5–32.2)	0.115	22.1 (17.5–27.6)	30.5 (22.2–40.4)	0.105
Administering drugs with the purpose of ending the patient’s life	16.9 (13.4–21.2)	14.0 (8.2–22.8)	17.9 (13.8–22.9)	0.395	15.1 (11.3–20.0)	21.9 (14.8–31.1)	0.132

Answers to the question: “Which of the following approaches would you consider an acceptable way of limiting intensive care in selected circumstances?”. Percentages of respondents who answered “acceptable” with 95% confidence intervals are shown. Answers are listed in decreasing acceptance. Total *n* = 396

### Parental involvement in decision-making

More than half of the respondents (60%) thought that parents should have the opportunity to take part in the decision-making process; this opinion was shared more often in the German speaking than in the French speaking area (64% vs. 49%). Moreover, 15% thought that parents should always have the opportunity to decide the course of action for their infant. Nurses (25%), as compared to physicians (12%), were more in favour of not directly involving parents in decision-making; they indicated that parental wishes and attitudes should be explored indirectly and considered by the decision-making health care team (25% vs. 12%). Only 0.5% of all HCPs thought that parents should not be involved at all, but merely be informed about the decision (see Additional file 2).

HCPs gave several reasons for not directly involving parents in decisions on treatment limitations (Table 4). Firstly, 95% considered that parents should not be involved because they might change their minds later and experience feelings of guilt. Secondly, they felt that parents should be spared the burden of such decisions (90%), with significantly more nurses taking that standpoint than neonatologists (94% vs. 71%). Thirdly, parents might not be in the right state of mind to take such decisions (76%), again with more nurses agreeing with that view (81% vs. 50%). Finally, nurses more often than physicians (82% vs. 36%) stated that parents canot fully understand the possible options and consequences (74%). Interestingly, 47% of HCPs took up the view that the responsibility for such decisions belongs solely to the physicians (nurses 47% vs. 46% physicians, ns); this position was significantly more often cited in the French speaking than in the German speaking area (60% vs. 37%).

**Table 4:** Reasons why parents should not directly be involved in decision-making

	Total	Physicians	Nurses	*p*-value	German speaking area	French speaking area	*p*-value
Parents might change their minds later and feel guilty	95.4 (88.8–98.2)	92.9 (68.5–98.7)	95.9 (88.6–98.6)	0.622	98.0 (89.7–99.7)	91.7 (78.2–97.1)	0.165
Parents should be spared the burden of such decisions	90.1 (82.3–94.7)	71.4 (45.4–88.3)	93.5 (85.7–97.2)	0.011	84.9 (72.9–92.1)	97.4 (86.5–99.5)	0.051
Parents are not in the right state of mind to take such decisions	76.2 (66.1–84.0)	50.0 (26.8–73.2)	81.4 (70.8–88.8)	0.012	73.9 (59.7–84.4)	78.9 (63.7–88.9)	0.592
Parents cannot fully understand the possible options and consequences	74.1 (63.9–82.2)	35.7 (16.3–61.2)	81.7 (71.2–89.0)	< 0.001	66.7 (52.5–78.3)	83.8 (68.9–92.3)	0.076
The responsibility for such decisions belongs solely to the physician	46.5 (36.3–57.0)	46.2 (23.2–70.9)	46.6 (35.6–57.9)	0.978	36.7 (24.7–50.7)	59.5 (43.5–73.7)	0.038
Parents might change their minds later and sue the physician	35.6 (25.6–47.1)	16.7 (4.7–44.8)	39.3 (28.1–51.9)	0.136	37.2 (24.4–52.1)	33.3 (19.2–51.2)	0.735
Discussing options of limiting care may jeopardize the trust parents have in the health care providers	23.1 (15.1–33.6)	15.4 (4.3–42.2)	24.6 (15.8–36.3)	0.474	32.6 (20.5–47.5)	11.4 (4.5–26.0)	0.029
Once involved, parents may become intrusive and put inappropriate pressure on the staff	20.3 (12.9–30.4)	16.7 (4.7–44.8)	20.9 (12.9–32.1)	0.739	13.3 (6.3–26.2)	29.4 (16.8–46.2)	0.080

Answers to the question: “For which of the following reasons should parents not be directly involved in the decision about whether or not to limit intensive care?” Percentages of respondents who answered “yes” with 95% confidence intervals are shown. Answers are listed in decreasing agreement. Total *n* = 91

### Disagreement between HCPs and parents

**Fig. 1:**
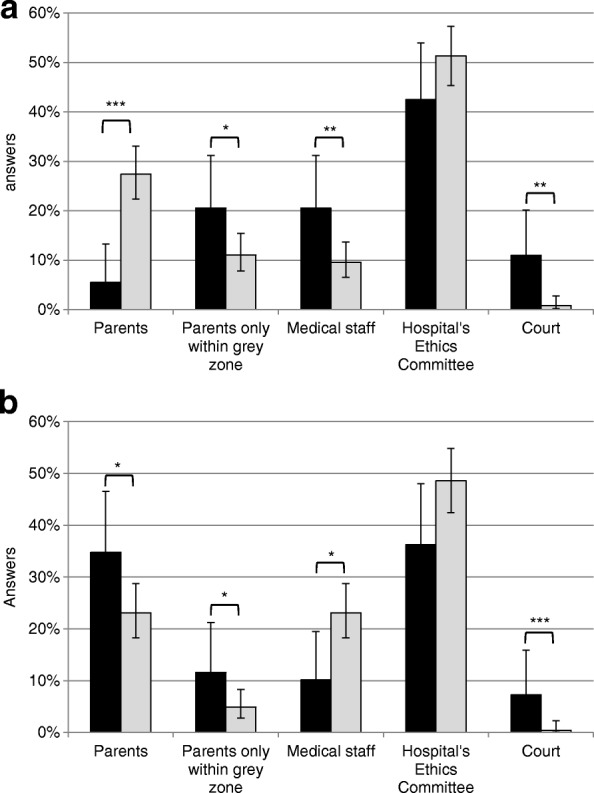
Ultimate decision-maker in cases of disagreement between parents and neonatal HCPs. **a** Answers to the question: “If parents request a limitation of intensive care, while the neonatal team thinks that treatment should be continued, who should be the ultimate decision-maker?” (*n* = 73 physicians, *n* = 263 nurses). **b** Answers to the question: “If parents request a continuation of intensive care, while the neonatal team thinks that treatment should be suspended, who should be the ultimate decision-maker?” (*n* = 69 physicians, *n* = 247 nurses). Percentages of valid answers with 95% confidence intervals are shown. Black bars represent physicians, grey bars nurses. * *p* < 0.05, ** *p* < 0.01, *** *p* < 0.001

In the event that parents would request limitation of intensive care, while HCPs recommend continuation of treatment, 43% of respondents considered hospital ethics committees to be the ultimate decision-makers and 31% felt that this would be the right of the parents. Another 10% indicated the medical staff and only very few (3%) chose the court as ultimate decision-makers (Fig 1a). In the opposite situation, namely if the parents request continuation of intensive care, while HCPs think that life-sustaining therapies should be withdrawn, 36% of all respondents considered the ethics committee to be the ultimate decision-maker, 20% named the parents, 16% the medical staff and only 1.5% the court (Fig. 1b). Nurses differed significantly from physicians in both scenarios.

### Hospital’s ethics committee

The role of hospital’s ethics committee was seen as follows: 80% of respondents indicated to give advice in individual cases, 12% being responsible for ultimate decision-making in individual cases - the latter was favoured more often by nurses (14%) than by physicians (6%) - and 5% only to set general guidelines; this view was taken more often in the German speaking area (6% vs. 0.9% in the French speaking area) (see Additional file 3).

### Prenatal decision-making

**Fig. 2:**
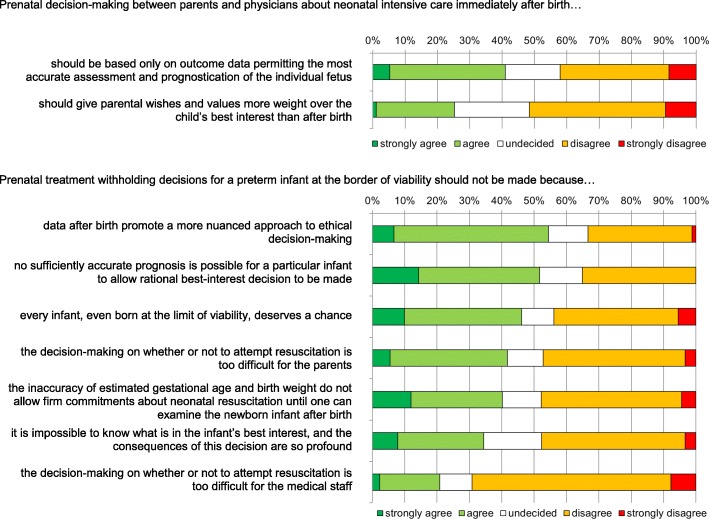
Difficulties in prenatal decision-making about neonatal intensive care immediately after birth. Answers are listed in decreasing agreement. *n* = 96 (physicians only)

A majority of neonatologists argued that decisions to withhold life-sustaining therapies from infants born at the limit of viability should not be made prenatally because prognosis is not sufficiently accurate (52%) and individual assessment after birth allows a more nuanced approach (54%) (Fig 2). There was no consensus among physicians whether parental wishes and the child’s best interest should be viewed differently before than after birth: 25% of physicians agreed that, prenatally, parental wishes and values should be given more weight than the child’s best interest, whereas this no longer is true after birth; 51% disagreed with this statement.

## Discussion

For the interpretation of these results, it is important to note that Switzerland is a diverse country with four official languages and regions, in which cultural differences are often reported [12]. In contrast to what we expected, our study found only few differences between the German- and French-speaking regions of Switzerland. One reason may be the fact that more than 50 % of the HCP working in Swiss NICUs come from other countries [13]. Therefore, the differences between language regions are blurred. Professional experience and religion also had little influence on the answers given.

Social cohesion and solidarity are important values in Switzerland [14]. Interestingly, nurses in our study considered society’s lack of solidarity and shortage of services for disabled as problematic within the decision-making process. This stands in contrast to a Swiss population survey about extreme prematurity, where a large majority of the population expressed substantial solidarity towards disabled people and did not perceive a decrease of solidarity over the last years [15]. More importantly, extremely premature infants born in Switzerland are provided with high quality care in which long-term economic considerations should not interfere with ethical decision-making for an individual case [16].

Traditionally, Swiss health care professionals had a more restrictive attitude towards providing proactive care for infants born at the limit of viability [3]. At present, the ideal decision-making model is considered a collaborative approach where parents and the health care team together make decisions regarding the treatment of an extreme premature infant [17]. In Switzerland, this is currently in development. For instance, one national study showed that decisions regarding resuscitation were less often shared in the neonatal unit [18]. Another study on decision-making in Switzerland showed how one perinatal centre was in the midst of changing from an informed consent approach to a shared approach [19]. Hence, overall, the development of a shared approach in Switzerland is still underway and practices vary enormously [20].

### Discrepancies between nurses and physicians

This survey reveals important differences between nurses and physicians regarding end-of-life decision-making in extremely preterm infants. These differences concern the perceived importance of specific challenges in the decision-making process, of acceptable approaches to limiting intensive care and of the degree of parental involvement in the decision-making process. For instance, nurses considered insufficient time for decision-making, legal constraints and lack of a consistent unit policy significantly more often problematic than physicians did. Moreover and although the large majority of neonatologists and neonatal nurses agree that withholding intensive care is acceptable in futile situations, nurses were more reluctant to withdraw respiratory support and tube feeding. Several speculations can be put forward when trying to explain these discrepancies between neonatal nurses and physicians.

First, differences may exist concerning education in medical ethics and ethical arguing. In Switzerland, physicians traditionally had a longer and perhaps more intensive training and exposure to ethical dilemmas, starting at medical school and continuing into professional life. Over the last years, medical ethics have also been incorporated into the curriculum of neonatal nurses and most units provide a continued training including ethical topics such as futility, over- and undertreatment, withholding and withdrawing lifesaving interventions.

Second, the differing views may be due to the different roles physicians and nurses play within the end-of-life decision process of extremely preterm infants [21]. Physicians are expected to have an understanding of the prognostic and outcome data of infants born extremely premature. Often considered less emotionally attached to the patient as nurses, they provide ‘an expert’ point of view to the team and parents. This medical judgement however is encumbered with significant medical prognostic uncertainty. It is therefore no surprise that physicians rather than nurses indicate prognostic issues as challenging. The active involvement of neonatal nurses in end-of-life decisions in NICUs was implemented only in recent years [22, 23]. Prior to that, the opinion of the nursing staff was not, or not explicitly, taken into account [24].

Third, the discomfort reported by nurses regarding some forms of therapy limitations (e.g. withdraw tube feeding, refraining from increasing respiratory parameters) may stem from the fact that the nurses will be more directly exposed to the patients and the parents. In some Swiss NICUs, a framework for decision-making has been introduced and has shown to reduce stress for both, physicians and nurses [25].

### Parental involvement in decision-making

The results of our survey indicate that today the Swiss HCPs are more willing to involve parents in the decision-making process than 10 years ago; however, they still do not give them full authority [26]. The trend towards increasing parental involvement in end-of-life decisions has also been reported in other countries [27–29].

The Swiss Civil Code for the protection of adults and children, enacted in 2013, emphasises parental authority when a child is incapable of making his or her own decisions. Shared decision-making between HCPs and parents is proposed by many national guidelines [7, 16]. It is a process aiming at a collaborative decision. This process may not be easy and disagreement, rarely reported in the past, has become more frequent in recent times [30]. Alandagady et al. observed that parents quite often do not agree with HCPs on limitations of life-sustaining therapies [31]. In this context, it is noteworthy, that in our study, HCPs give more weight to parental opinions in cases of disagreement when parents request limitation of intensive care compared to situations where they ask for continuation of intensive care. Although the difference between withholding and withdrawing intensive care measures are morally equivalent, these differences might be considered relevant in practice [32]. In both cases, a large percentage of respondents think that a hospital’s ethics committee should be the ultimate decision-maker. This stands in contrast to a Swiss population survey, where a large majority of lay-people expressed that parents should be the final decision-makers [12].

There are differences between nurses and physicians in how and to which extent parents should be involved in the decision-making process. Nurses were more in favour of integrating the views of the parents indirectly rather than to directly confront them with the choice between several options and to embark in a shared decision-making. In Switzerland, neonatal nurses are fully and directly involved in the decision-making process, together with the physicians and parents. However, it could be that they are more prone to embrace the ‘expert opinion’ of physicians regarding the prognosis and outcome for a given infant than to incorporate parental values, which often include aspects of family autonomy, parental obligations towards other children and own legitimate self-interests. As stated by Leuthner, the concept of best interest and medical expertise on the course of neonatal diseases and outcome data is a too restrictive concept of decision-making [32]. Only the addition of parental values gives meaning to the prognosis. Moreover, within the triad physicians, nurses and parents, it is often the case that over the first days and weeks when it may come to a decision-making process, the nurses have the most close and intimate contact with the premature infant. This fact may add to the greater difficulty for neonatal nurses to acknowledge and integrate parental values and attitudes within the decision-making process.

HCPs who believed parents should not be (directly) involved in the decision-making on whether or not to limit intensive care, gave several reasons for doing so. These reasons, however, are empirically unfounded or proven false. For example, Caymeax et al. showed that greater involvement of parents in end-of-life decisions was associated with lower levels of grief [33].

### Prenatal decision-making

Prenatal decisions differ in several aspects from decisions made after birth not only because the legal status of an unborn child differs from that of a liveborn infant, but also because the prognosis is much more uncertain [34, 35]. Therefore, a prenatal decision not to resuscitate an infant born at the limit of viability should be based on strict criteria and take into account the wishes and preferences of the parents [6, 36].

#### Strength of this study

The high response rate of motivated HCPs is likely to result in a representative nationwide assessment of opinions of HCPs regarding end-of-life decision-making. The large sample size allows subgroup analyses. Since several identical questions as in previous surveys were used, time and societal trends and differences to other countries can be assessed.

#### Limitations of this study

The answers given online are self-reported qualitative judgments that are difficult to quantify. The number of questions had to be limited to avoid impeding participation and, therefore, several aspects could not be explored in more depth.

## Conclusions

Physicians and nurses differ in many aspects of how and by whom end-of-life decisions should be made in extremely preterm infants. Acknowledging these differences is important to avoid potential conflicts within the neonatal team but also with parents in the process of end-of-life decision-making in preterm infants born at the limits of viability.

## Additional files

Additional file 1: Full questionnaire. (PDF 121 kb)

Additional file 2: Involvement of parents. (DOCX 13 kb)

Additional file 3: Role of the Hospital’s Ethics Committee. (DOCX 15 kb)

## Abbreviations

HCPs: Health Care Professionals
NICU: Neonatal intensive care unit
