# Correction to: decision-making at the limit of viability: differing perceptions and opinions between neonatal physicians and nurses

**DOI:** 10.1186/s12887-018-1204-x

**Published:** 2018-07-09

**Authors:** Hans Ulrich Bucher, Sabine D. Klein, Manya J. Hendriks, Ruth Baumann-Hölzle, Thomas M. Berger, Jürg C. Streuli, Jean-Claude Fauchère, on behalf of the Swiss Neonatal End-of-Life Study Group, Philipp Meyer, Roland Neumann, Renate Itin, Mathias Nelle, Liliane Stoffel, Brigitte Scharrer, Kai Roloff, Riccardo Pfister, Matthias Roth-Kleiner, Magali Contino, Thomas M. Berger, Ulrike Schlegel, Gudrun Jaeger, Ruth Dutler, Jean-Claude Fauchère, Barbara Dinten

**Affiliations:** 1 Department of Neonatology, University Hospital Zurich, Frauenklinikstrasse 10, 8091 Zürich, Switzerland; 2 Institute of Biomedical Ethics and History of Medicine, University of Zurich, Zurich, Switzerland; 3 Dialogue Ethics Foundation, Interdisciplinary Institute for Ethics in Health Care, Zurich, Switzerland; 4 Neonatal and Paediatric Intensive Care Unit, Children’s Hospital of Lucerne, Lucerne, Switzerland

## Correction

After publication of the original article [1], the corresponding author noticed the given names and family names of the members included in the Swiss Neonatal End-of-Life Study Group were incorrectly reverted. The correct version of the Acknowledgements section has been included in this Correction and includes the correct presentation of names for the members included in the Swiss Neonatal End-of-Life Study Group.

## References

[1] Bucher HU (2018). Decision-making at the limit of viability: differing perceptions and opinions between neonatal physicians and nurses. BMC Pediatr.

