# Correction to: Decision-making at the limit of viability: differing perceptions and opinions between neonatal physicians and nurses

**DOI:** 10.1186/s12887-019-1890-z

**Published:** 2020-01-31

**Authors:** Hans Ulrich Bucher, Sabine D. Klein, Manya J. Hendriks, Ruth Baumann-Hölzle, Thomas M. Berger, Jürg C. Streuli, Jean-Claude Fauchère, on behalf of the Swiss Neonatal End-of-Life Study Group

**Affiliations:** 1 Department of Neonatology, University Hospital Zurich, Frauenklinikstrasse 10, 8091 Zürich, Switzerland; 2 Institute of Biomedical Ethics and History of Medicine, University of Zurich, Zurich, Switzerland; 3 Dialogue Ethics Foundation, Interdisciplinary Institute for Ethics in Health Care, Zurich, Switzerland; 4 Neonatal and Paediatric Intensive Care Unit, Children’s Hospital of Lucerne, Lucerne, Switzerland

**Correction to: BMC Pediatr (2018)**

**https://doi.org/10.1186/s12887-018-1204-x**

Please be advised that following publication of the original article [1], it was brought to the authors’ attention that they did not have permission to reproduce the questionnaire in Additional File 1, and so Additional File 1 has since been removed from the article.

In addition, please note that the following (required) statement was previously missing from the Acknowledgements of the article:

“We used the questionnaires developed for the EURONIC Project on ‘Parents’ information and ethical decision making in neonatal intensive care units: staff attitudes and opinions’ (EU Contract n. BMH1-CT93-1242) with permission. Requests for use of the questionnaires should be addressed to Marina Cuttini (marina.cuttini@opbg.net; marinacuttini@gmail.com).”

Furthermore, after publication of the original article [1], the corresponding author noticed that the given names and family names of the members included in the Swiss Neonatal End-of-Life Study Group had been erroneously reverted in the initial publication.

These errors have all been corrected in the original article.
