# Supplementary material for: Decision-making at the limit of viability: differing perceptions and opinions between neonatal physicians and nurses
Source: BMC Pediatr. 2018 Feb 22;18:81. doi: 10.1186/s12887-018-1040-z (PMC5822553; doi:10.1186/s12887-018-1040-z)
Supplement: Supplementary file 1 — Full questionnaire. (PDF 121 kb) [file 12887_2018_1040_MOESM1_ESM.pdf]

Readers are alerted that the Additional file 1 presented in this article has been removed due to a reported legal dispute. We are currently investigating these concerns, following which editorial action will be taken as appropriate.
