# Supplementary material for: Decision-making at the limit of viability: differing perceptions and opinions between neonatal physicians and nurses
Source: BMC Pediatr. 2018 Feb 22;18:81. doi: 10.1186/s12887-018-1040-z (PMC5822553; doi:10.1186/s12887-018-1040-z)
Supplement: Supplementary file 2 — Involvement of parents. (DOCX 13 kb) [file 12887_2018_1040_MOESM2_ESM.docx]

Additional file 2

Table 1. Involvement of parents.

|  | Total | Physicians | Nurses | p-value |
| --- | --- | --- | --- | --- |
| Yes, they should have the opportunity to choose the course of action for their baby in any case | 15.1 | 14.7 | 15.2 | 0.906 |
| Yes, they should have the opportunity to choose the course of action for their baby, but only within the grey zone | 2.3 | 4.2 | 1.7 | 0.145 |
| Yes, they should have the opportunity to take part in the decision | 59.7 | 66.3 | 57.6 | 0.132 |
| No, but their attitudes and wishes should be indirectly sounded out and taken into account | 21.4 | 11.6 | 24.5 | 0.007 |
| No, but they should be informed about the decision which is taken | 0.5 | 0.0 | 0.7 | 0.427 |
| Other | 1.0 | 3.2 | 0.3 | 0.016 |

|  | Total | German speaking area | French speaking area | p-value |
| --- | --- | --- | --- | --- |
| Yes, they should have the opportunity to choose the course of action for their baby in any case | 15.1 | 15.5 | 14.2 | 0.738 |
| Yes, they should have the opportunity to choose the course of action for their baby, but only within the grey zone | 2.3 | 1.8 | 3.5 | 0.283 |
| Yes, they should have the opportunity to take part in the decision | 59.7 | 64.1 | 48.7 | 0.005 |
| No, but their attitudes and wishes should be indirectly sounded out and taken into account | 21.4 | 17.6 | 31.0 | 0.003 |
| No, but they should be informed about the decision which is taken | 0.5 | 0.4 | 0.9 | 0.499 |
| Other | 1.0 | 0.7 | 1.8 | 0.338 |

Percentage of answers to the question: “If a decision about whether or not to limit intensive care for a baby is under consideration, should the parents be involved as a rule? (Please choose only one answer)” Total n = 397.
