# Supplementary material for: Decision-making at the limit of viability: differing perceptions and opinions between neonatal physicians and nurses
Source: BMC Pediatr. 2018 Feb 22;18:81. doi: 10.1186/s12887-018-1040-z (PMC5822553; doi:10.1186/s12887-018-1040-z)
Supplement: Supplementary file 3 — Role of the Hospital’s Ethics Committee. (DOCX 15 kb) [file 12887_2018_1040_MOESM3_ESM.docx]

Additional file 3

Table 2. Role of the Hospital’s Ethics Committee.

a)

|  | Total | Physicians | Nurses | p-value |
| --- | --- | --- | --- | --- |
| It should have no role | 1.3 | 1.1 | 1.3 | 0.829 |
| Its only role should be the setting of general guidelines, but not intervening in decisions regarding individual cases | 4.6 | 8.4 | 3.3 | 0.039 |
| It should have the role of giving advice on decisions regarding individual cases | 79.7 | 80.0 | 79.6 | 0.932 |
| It should be responsible for the ultimate decision regarding individual cases | 12.2 | 6.3 | 14.0 | 0.045 |
| Other | 2.3 | 4.2 | 1.7 | 0.150 |

|  | Total | German speaking area | French speaking area | p-value |
| --- | --- | --- | --- | --- |
| It should have no role | 1.3 | 1.4 | 0.9 | 0.666 |
| Its only role should be the setting of general guidelines, but not intervening in decisions regarding individual cases | 4.6 | 6.0 | 0.9 | 0.027 |
| It should have the role of giving advice on decisions regarding individual cases | 79.7 | 80.1 | 78.8 | 0.770 |
| It should be responsible for the ultimate decision regarding individual cases | 12.2 | 10.7 | 15.9 | 0.149 |
| Other | 2.3 | 1.8 | 3.5 | 0.291 |

Percentage of answers to the question: “What do you think the Hospital’s Ethics Committee’s role should be in making decisions about whether or not to limit intensive care? (Please choose only one answer)” Total n =394 (95 physicians, 299 nurses, 281 from German speaking area, 113 from French speaking area).

b)

|  | Total | Physicians | Nurses | p-value |
| --- | --- | --- | --- | --- |
| Always, when a decision about whether or not to limit intensive care for a patient is under consideration | 35.8 | 23.8 | 39.2 | 0.011 |
| When requested by parents and/or staff | 58.1 | 65.0 | 56.1 | 0.156 |
| Other | 6.1 | 11.3 | 4.7 | 0.031 |

|  | Total | German speaking area | French speaking area | p-value |
| --- | --- | --- | --- | --- |
| Always, when a decision about whether or not to limit intensive care for a patient is under consideration | 35.8 | 43.1 | 18.1 | <0.001 |
| When requested by parents and/or staff | 58.1 | 52.2 | 72.4 | <0.001 |
| Other | 6.1 | 4.7 | 9.5 | 0.086 |

Percentage of answers to the question: “When should the Hospital’s Ethics Committee be consulted for making decisions? (Please choose only one answer)” This question was only asked to respondents who chose answer 3 or 4 in the previous question. Total n = 358 (80 physicians, 278 nurses, 253 from German speaking area, 105 from French speaking area).
